# Phospholipase C-β1 Hypofunction in the Pathogenesis of Schizophrenia

**DOI:** 10.3389/fpsyt.2015.00159

**Published:** 2015-11-17

**Authors:** Seong-Wook Kim, Taesup Cho, Sukchan Lee

**Affiliations:** ^1^Center for Cognition and Sociality, Institute for Basic Science, Daejeon, South Korea; ^2^Department of Physiology, Seoul National University College of Medicine, Seoul, South Korea

**Keywords:** schizophrenia, PLC-**β**1, schizophrenia endophenotypes, mPFC, DLPFC

## Abstract

Schizophrenia is a mental disorder that is characterized by various abnormal symptoms. Previous studies indicate decreased expression of phospholipase C-β1 (PLC-β1) in the brains of patients with schizophrenia. PLC-β1-null (PLC-β1^−/−^) mice exhibit multiple endophenotypes of schizophrenia. Furthermore, a study of PLC-β1 knockdown in the medial prefrontal cortex of mice has shown a specific behavioral deficit, impaired working memory. These results support the notion that disruption of PLC-β1-linked signaling in the brain is strongly involved in the pathogenesis of schizophrenia. In this review, we broadly investigate recent studies regarding schizophrenia-related behaviors as well as their various clinical and biological correlates in PLC-β1^−/−^ and knockdown mouse models. This will provide a better understanding of the pathological relevance of the altered expression of PLC-β1 in the brains of patients with schizophrenia. Evidence accumulated will shed light on future in-depth studies, possibly in human subjects.

## Introduction

### Schizophrenia-Related Phenotypes

Schizophrenia is generally characterized by genetic and neurofunctional abnormalities and classified into three major abnormal symptoms – positive, negative, and cognitive symptoms (1–6). Positive symptoms are those that are present in people with schizophrenia, such as delusions, hallucinations, thought disorder, and paranoia (7), whereas negative symptoms are deficits of normal emotional or of other thought processes such as flat affect, avolition, and social withdrawal (8). Cognitive symptoms refer to the difficulties with concentration and memory; examples include short- and long-term memory deficits as well as deficits in attention, planning, and abstract thinking (9–11).

### Pathogenesis of Schizophrenia

The pathogenesis of schizophrenia has been previously reported to arise from specific neuronal abnormalities in several brain regions including prefrontal cortex (PFC) (12–14). Neural mechanisms underlying schizophrenia symptoms in several brain areas have been explained by abnormalities in the dopaminergic (15, 16), serotonergic (17, 18), muscarinic (19–22), and glutamatergic signaling (23–25). Of the many mechanisms, the phosphoinositide (PI) signaling, one of the major G-protein-linked pathways operating in the central nervous system (CNS), seems to be a point of convergence for all signaling pathways mentioned above (26, 27). PI signaling pathways are impaired in specific brain regions of patients with neurological (28–31) and psychiatric disorders (32, 33). Altered activity in PI signal pathways has been implicated in impaired cognition, mood, and abnormal behaviors, which is associated with mental disorders including schizophrenia (34, 35).

### PI Signaling

**Figure 1:**
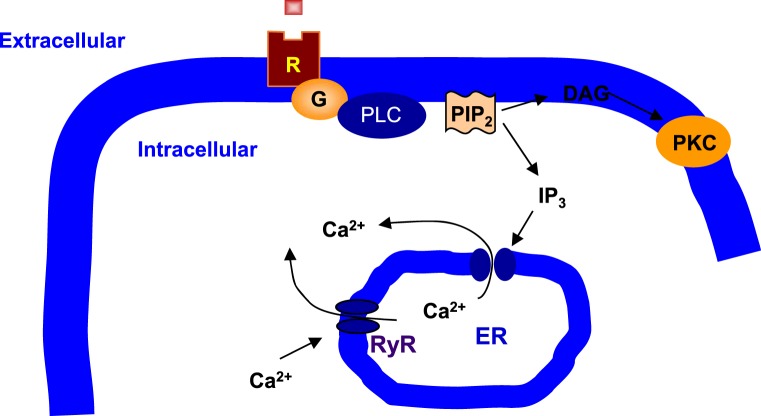
**Phospholipase C signaling in neuronal cells**. Phospholipase C (PLC) cleaves phosphatidylinositol 4,5-bisphosphate (PIP_2_), a membrane phospholipid, to generate two second messengers, inositol 1,4,5-trisphosphate (IP_3_) and diacylglycerol (DAG). IP_3_ is water soluble, diffusing through the cytosol to bind to and open a ligand-gated Ca^2+^ channel, such as ryanodine receptor (RyR) in the endoplasmic reticulum (ER). Thus, stimulation of a receptor (R) linked to this G-alpha (G) is a way to increase Ca^2+^ inside the cytosol. DAG is lipid soluble and stays in the membrane. It activates protein kinase C (PKC), which, like protein kinase A (PKA), phosphorylates particular target proteins.

PI signaling involves PI-specific phospholipase C (PLC) (36, 37) (Figure 1). PLC hydrolyzes phosphatidylinositol 4,5-bisphosphate (PIP_2_) to produce a pair of second messengers, diacylglycerol (DAG), and inositol 1,4,5-trisphosphate (IP_3_) (38). In general, DAG activates protein kinase C (PKC), whereas IP_3_ mobilizes Ca^2+^ from the intracellular endoplasmic reticulum (ER) stores to the cytoplasm (39). PLC-β is one of the three subtypes of PLC and is distinguished from PLC-γ and PLC-δ by structure and activation mechanisms (40, 41). PLC-β acts through G protein-dependent pathways, and G_q_/PLC-β pathway is readily activated by specific neurotransmitter receptors such as the M1, M3, and M5 muscarinic acetylcholine receptors (mAChRs), the group 1 and 5 metabotropic glutamate receptors (mGluRs), and the 2A and 2C subtype of serotonergic receptors (5-HT_2A_R and 5-HT_2C_R) (42–46). Protein purification and molecular cloning have identified four PLC-β isoforms: PLC-β1, PLC-β2, PLC-β3, and PLC-β4 (47, 48). Interestingly, each PLC-β isoenzyme has a unique distribution pattern in the brain (49). PLC-β1 expression is relatively abundant in the cerebral cortex although it is widely distributed in many brain areas (45, 49). PLC-β2 is mainly expressed in the white matter, indicating that its expression is in non-neuronal cells (49). PLC-β3 expression is very low throughout the brain (50). Finally, PLC-β4 is highly expressed in the cerebellum and medial septum but is almost negligible in the hippocampus (49–51).

Abnormal PLC-β1 expression has been detected in the brains of patients with schizophrenia (52–54). A decrease of PLC-β1 expression in the dorsolateral prefrontal cortex (DLPFC) of the brains of patients with schizophrenia might account for the possible pathogenic involvement of PLC-β1 in schizophrenia patients (54).

In this review, PLC-β1 signaling pathways, especially their hypofunction will be discussed in relation to the pathogenesis of schizophrenia-like symptoms in the rodent medial prefrontal cortex (mPFC) as well as their hypofunction in patients with schizophrenia associated with human DLPFC.

## PLC-β1 in Schizophrenia Symptoms

The association of the PLC-β1 with schizophrenia symptoms has been addressed by behavioral studies in PLC-β1^−/−^ mice (55–59). It has been well established that schizophrenia-like animal models exhibit hyperlocomotion (56, 57, 60–64). In the open-field test, for example, PLC–β1^−/−^ mice have shown an increase in locomotor activity (56, 57), a phenotype which was also exhibited in mice with a defect in PLC–β1-relevant signaling. For instance, M1 mAChR knockout results in hyperlocomotion in mice (63, 64), whereas mice lacking M3 and M5 mAChRs show normal locomotor activity (65, 66).

Abnormal social behaviors are regarded as one of the negative symptoms in schizophrenia patients. In mice, barbering behavior, also known as whisker trimming, seems to reflect a cooperative social activity and social dominance (67). Interestingly, PLC–β1^−/−^ mice show a deficit in barbering behaviors, suggesting that PLC–β1 signaling is required for normal social interaction and dominance (56). In addition, the relevance of the lack of nesting behaviors to negative symptoms in patients with schizophrenia has been demonstrated (68, 69). Nest building in mice may represent activities of daily living (70) and a cooperative activity in rodent social behavior (68). Deficits in nest building are related to self-neglect (56) and social withdrawal (71). PLC–β1^−/−^ mice also display significant nesting deficits, addressing negative-like symptoms in rodents.

The lack of sociability is also regarded as a negative symptom in schizophrenia patients (72, 73). A three-chamber procedure was previously used to evaluate sociability by assessing the time spent in each chamber of three divided areas. One area contains a conspecific, but the other area does not. If a mouse spends more time in the chamber with a conspecific, it has higher sociability (74). This procedure has been complemented by measuring sniffing time because sniffing time is more accurate than time in chamber when evaluating sociability (59). The total sniffing time around either a mouse or an object was scored. If the time spent sniffing around an unfamiliar conspecific was greater than an inanimate object, this is indicative of higher sociability. PLC-β1^+/+^ mice showed normal sociability as they spent more time sniffing around an unfamiliar conspecific than an inanimate object. On the contrary, sniffing time around both a conspecific and inanimate object was decreased in PLC-β1^−/−^ mice. Consequently, PLC-β1^−/−^ mice have lost their interest in the novelty of an unfamiliar conspecific, demonstrating impaired sociability (59).

Cognitive impairment is a core feature of three abnormal symptoms that contribute to the morbidity of schizophrenia. Of cognitive symptoms, sensorimotor gating is essentially a protection mechanism against sensory information overload (75). A disruption of prepulse inhibition (PPI) is a frequently used translational model of abnormal sensorimotor gating (76). The PPI deficits have been detected in some neuropsychiatric disorders including schizophrenia (77–84). Therefore, PPI may at least in part be predictive of certain cognitive functions in schizophrenia (84). Furthermore, PLC-β1^−/−^ mice also display impaired PPI in acoustic startle response (56, 57). Given that schizophrenia patients and PLC-β1^−/−^ mice show impairment in PPI, a measure of sensorimotor gating, PLC-β1 signaling pathways seem to be required for normal sensorimotor gating function.

Impaired working memory is considered as a core cognitive deficit in schizophrenia (85, 86). Delayed non-match to samples (DNMTS) T-maze and Y-maze task have been commonly used to measure working memory function (87, 88). PLC-β1^−/−^ mice exhibit an impairment of working memory in the DNMTS T-maze and Y-maze task (56, 58). Based on the working memory deficits in PLC-β1^−/−^, PLC-β1 may be a potential target of pharmaceutical intervention to treat cognitive symptoms in schizophrenia.

## Human DLPFC and Rodent mPFC

Working memory deficits have been previously reported in a number of neurological and psychiatric disorders including schizophrenia (89–91). Previous studies from human behavioral and functional neuroimaging data during working memory tasks have described deteriorated activation of the DLPFC in patients with schizophrenia (92–94). The human DLPFC, therefore, is important for the working memory process (95, 96). Signals associated with working memory are reduced in the DLPFC of patients with schizophrenia (97, 98). Additionally, deficits in working memory have been shown in human brain injury patients with damage in DLPFC (99, 100). A decrease of both glucose utilization and blood flow during working memory tasks have been observed in the DLPFC of patients with schizophrenia (94, 101–104). Using electroencephalography (EEG) during a working memory test, abnormal brain oscillatory activity has been reported in the frontal theta (4–8 Hz) and alpha (8–12 Hz) frequencies in patients with schizophrenia (105, 106). Human EEG recordings have further revealed abnormal circuitries between the PFC and other brain regions in schizophrenia, such as the temporal lobe and subcortical limbic structures, suggesting that functional connectivity between the PFC and other brain regions play some role in the pathogenesis of schizophrenia (107–109). It has been supported by evidence that alterations in synchronized brain oscillation reflect neuronal changes that lead to schizophrenia (110, 111).

The mouse mPFC is thought to have anatomical and functional homology with the DLPFC in human being (38, 112–114). The mouse mPFC consists of prelimbic (PL) and infralimbic (IL) cortices (38, 112–114). The mouse mPFC receives indirect projections from the dorsal hippocampus (dHPC) and direct afferent inputs from the ventral hippocampus (vHPC) (115, 116). The mouse mPFC has reciprocal connections with the amygdala and other subcortical limbic structures (117). Theta frequency synchrony between the mPFC and dHPC, and/or beta (13–30 Hz) frequency synchrony between the mPFC and mediodorsal thalamic nucleus (MD) are required for working memory (13, 118).

Both cytotoxic lesions and acute inactivations in the rodent mPFC are able to induce most schizophrenia phenotypes including positive, negative, and cognitive-like symptoms, as well as working memory deficits (119, 120). It is also worth noting that mPFC deficits represent a key component of the pathophysiology in patients with schizophrenia (14, 121).

The shRNA-mediated silencing of PLC-β1 in the mPFC, a mouse model that mimics the decrease of PLC-β1 in the DLPFC of patients with schizophrenia, causes an impairment in working memory (59). This effect is specific to working memory; mPFC-specific PLC-β1 knockdown does not have an effect on other behaviors relevant to schizophrenia-related endophenotypes characteristic of PLC-β1^−/−^ mice, such as locomotion, social behaviors, and sensorimotor gating (59).

## PLC-β1 Signaling Pathways Underlying Working Memory in the mPFC

A number of biochemical and genetic studies have demonstrated that modulatory neurotransmission in the PFC is required for cognitive functions including working memory (11, 12, 96, 122–125). Dopaminergic neurotransmission in the PFC has been shown to be important for working memory in both animals and human beings (126–134). Regional depletion of PFC dopamine causes profound working memory impairments in monkeys (126) and rats (127). Iontophoretic application of dopamine into the PFC for working memory tasks has revealed an increase of delay-period activity in monkeys (134). During working memory, the dopamine level is transiently increased in the PFC of both rats and human beings (135, 136). Explained briefly, dopamine receptors are divided into two families (D1 and D2 class) based on second messenger coupling and ligand binding (137, 138); D1 class, composed of D1 and D5 dopamine receptors, increases the cAMP levels, whereas D2 class, composed of D2, D3, and D4 receptors, decreases cAMP levels (139, 140). The influence of dopamine in prefrontal neurons is largely mediated by D1 dopamine receptors as they are much more abundant compared to D2 dopamine receptors in PFC pyramidal cells (141). The PFC D1 dopamine receptors are decreased in patients with schizophrenia (133). A D1 antagonist is able to suppress PFC delay-period activity. Similarly, the infusion of D1-specific agonists has further confirmed the importance of D1 dopamine receptors in the PFC with improved working memory (125, 142). Furthermore, the importance of D1 dopamine receptors has been investigated in non-human primates during delayed-response paradigms showing that working memory requires appropriate D1 receptor activation in the DLPFC (126, 129, 130, 142, 143). Taken together, these observations suggest that altered dopamine transmission at D1 receptors in DLPFC could be involved in the pathophysiology of working memory in schizophrenia (90, 95, 144, 145).

In addition to dopamine receptors involved in working memory, other modulatory neurotransmitters, such as norepinephrine (NE) acting through α-adrenergic receptors (146, 147), serotonin through 5-HT_2A_ receptors (124), and acetylcholine through mAChRs (148) have been shown to be involved in working memory. Moderate levels of NE activate α_2A_ adrenergic receptors and result in improved working memory; however, at higher levels, NE activates α_1_ receptors, resulting in impaired working memory (123, 149, 150). Similarly, it has been proposed that excessive prefrontal NE levels may contribute to the working memory deficits in schizophrenia (122). Investigation of M1 mAChRs knockout mice has provided evidence that M1 mAChRs are critical for the performance of non-matching-to-sample working memory tasks (151).

Several lines of research have indirectly indicated that Gq signaling pathways are implicated in working memory (12, 59, 124, 130). In detail, translocation of the α subunit of Gq proteins from the membrane to a cytosolic fraction can be used as an indicator of activation (152). Gq translocates during the delay period of the match-to-place task for working memory (125), suggesting that the Gq signaling cascade is activated during working memory. Consistently, alterations in prefrontal Gq signalings have been associated with working memory deficits in patients with schizophrenia (153). RGS4, an inhibitor of Gq protein-induced intracellular Ca^2+^ release, is downregulated in PFC of schizophrenia mouse model (58, 154).

## Conclusion and Future Remarks

Although continued investigation is required to fully understand PLC-β1 hypofunction in the pathogenesis of schizophrenia, the results obtained to date suggest some challenges in the treatments for working memory deficits in schizophrenia. As indicated previously, altered expression of PLC-β1 has been detected in several brain regions of patients with schizophrenia including DLPFC (52, 53, 155–158). The major schizophrenia symptoms (1–6), such as negative and cognitive symptoms, have been observed in the behavioral characterization of PLC-β1^−/−^ mice. These results indicate that disrupted PLC-β1 signaling in specific brain regions can be relevant to the pathogenesis of schizophrenia (55–58).

Furthermore, neurotransmitter transmissions such as dopamine through D1 receptors coupled to PLC in the mPFC (129, 141, 159, 160), NE acting through α-adrenoreceptors (146, 147), serotonin through 5-HT_2A_ receptors (124), and acetylcholine through M1 mAChRs (148) are necessary for working memory. The activities of intracellular second messenger pathways linked with PLC in the mPFC are also critical for working memory (161–163). In addition, PLC-β1 knockdown and impaired working memory in the mPFC have been discussed in this review, suggesting that the mPFC-specific PLC-β1 pathways underlying working memory could be different from those for anxiety or other schizophrenia endophenotypes of PLC-β1^−/−^ mice (59).

In conclusion, these results support the notion that the decrease in PLC-β1 expression in the brains of patients with schizophrenia is a pathogenically relevant molecular marker of the disorder (54, 56–59). This interpretation offers new insight into PLC-β1 hypofunction in the pathogenesis of schizophrenia and may aid in a better understanding of the neural mechanisms underlying working memory deficits in schizophrenia. Finally, PLC-β1 is not detected in a peripheral area (164, 165). Thus, PLC-β1 KO could not affect obesity or weight gain. PLC-β1 KO mice are even not hypophagic and lean unlike M3 KO mice (65). Therefore, the PLC-β1-based treatments are necessary for the development of novel psychotherapeutic approaches with reducing metabolic side effects.

## Conflict of Interest Statement

The authors declare that the research was conducted in the absence of any commercial or financial relationships that could be construed as a potential conflict of interest.
